# High Expression of Lewis y Antigen and CD44 Is Correlated with Resistance to Chemotherapy in Epithelial Ovarian Cancers

**DOI:** 10.1371/journal.pone.0057250

**Published:** 2013-02-28

**Authors:** Zhenhua Hu, Jian Gao, Danye Zhang, Qing Liu, Limei Yan, Lili Gao, Juanjuan Liu, Dawo Liu, Shulan Zhang, Bei Lin

**Affiliations:** Department of Obstetrics and Gynecology, Shengjing Hospital Affiliated to China Medical University, Shenyang, Liaoning Province, China; Baylor College of Medicine, United States of America

## Abstract

**Objectives:**

To measure Lewis y antigen and CD44 antigen expression in epithelial ovarian carcinoma and to correlate the levels of these antigens with clinical response to chemotherapy.

**Methods:**

The study cases included 34 cases of ovarian carcinoma with resistance to chemotherapeutic drugs, 6 partially drug-sensitive cases, and 52 drug-sensitive cases (92 total).

**Results:**

The rates of expression of Lewis y antigen and CD44 antigen were significantly greater in the drug-resistant group than that in the partially-sensitive or sensitive groups. Surgical stage, residual tumor size and expression of CD44 and Lewis y antigen in ovarian carcinoma tissues were independent risk factors for chemotherapeutic drug resistance.

**Conclusions:**

Over-expression of Lewis y and CD44 antigen are strong risk factors for chemotherapeutic drug resistance in ovarian carcinoma patients.

## Introduction

Development of resistance to chemotherapy in epithelial ovarian cancers involves multiple mechanisms. In recent years, cell adhesion-mediated drug resistance (CAM-DR) has become an active area of investigation in the study of tumor drug resistance. For example, the hyaluronan (HA)-specific receptor, CD44, which plays an important role in adhesion, has been shown to be involved in CAM-DR through interactions with its ligand HA [1], [2]. Several studies have revealed that CD44s is highly expressed in situ and metastatic ovarian cancers and that CD44 expression is correlated with malignant behaviors of ovarian cancer cells, such as adhesion, invasion, and metastasis [3], [4]. CD44 is modified post-translationally by glycosylation, which has been shown to influence CD44-mediated CAM-DR [5]. In our preliminary studies, we have found that, although CD44 mRNA levels were similar in α1,2-fucosyltransferase transfected ovarian cancer cells (RMG-1-H) and the parent cells (RMG-1), CD44 protein expression levels were significantly higher in the RMG-1-H cells. In addition, we found that the di-fucosylated Lewis y antigen was a part of the composition of CD44 and that increased expression of this antigen correlated with increased CD44-mediated ovarian cell adhesion and migration [6]. Increased expression of Lewis y antigen and CD44 in RMG-1-H cells was associated with increased resistance to chemotherapeutic drugs, including 5-fluorouracil, carboplatin and paclitaxel (Taxol®) [7], [8]. Although the effects of alternative splicing and post-translational glycosylation of CD44 on its interaction with HA have been widely studied in recent years, few reports have described the effects of alterations in fucosylation on CD44-dependent CAM-DR of ovarian cancer cells. Therefore, to address this question, in the present study, we have quantified ovarian cancer drug resistance and expression of Lewis y antigen and CD44 in tissues from ovarian cancer patients. We then used these data to investigate correlations between expression of Lewis y antigen and CD44 and chemotherapeutic resistance, in addition to assess the clinical significance of these correlations.

## Materials and Methods

This study was approved by the Institutional Review Board of Shengjing Hospital. Between May 2005 and July 2009, 92 Chinese patients diagnosed with primary epithelial ovarian cancer by surgery and pathological analysis were retrospectively identified from the records of Shengjing Hospital Affiliated to China Medical University. All patients underwent treatment for ovarian cancer that included surgical debulking, followed by 6–8 postoperative cycles of paclitaxel (Taxol®) plus carboplatin (TC regimen) conventional chemotherapy. The chemotherapy schedule was designed on the day of the surgery. Patients were followed for a minimum of one year after completion of chemotherapy. Patients' information, including age, surgical stage, grade, pathological subtype, lymph node metastasis and residual tumor size, were collected from clinical and pathological records (**Table 1**). Tumor biomarker levels and results from imaging and gynecological examinations were also recorded at the time of chemotherapy and at reexamination. The associated clinical data and chemotherapy follow-up data (≥2 years) for all patients was complete.

**Table 1:** Clinical And Tumor Characteristics.

		Drug-resistant (n = 34)	Partially sensitive (n = 6)	Sensitive (n = 52)	*P*-value
**Ages** (median, mean)years		55, 56.31	63, 62.67	54, 54.58	0.06
**Surgical Stage**					
I∼II stage		4	1	27	0.002
III∼VI stage		30	5	25	
**Tumor Grade**					
I		4	0	11	0.053
II		10	4	23	
III		20	2	18	
**Lymph Node Metastasis**					
Yes		8	1	4	0.004
No		9	2	36	
Unknown		17	3	12	
**Residual Tumor Size**					
≤1 cm		6	2	35	<0.0001
1∼2 cm		7	2	7	
≥2 cm		10	0	2	
**Group**		34	6	52	
**Lewis y**					
negative	−	3	3	20	
Positive	+	4	2	14	
	++	19	1	18	
	+++	8	0	0	
Positive ratio (%)		91.17(31/34)*	50.00(3/6)**	61.54(32/52)***	* *P*<0.01,** *P*<0.05,*** *P* = 0.915
**CD44**					
negative	−	3	2	25	
Positive	+	7	3	12	
	++	22	1	14	
	+++	2	0	1	
Positive ratio (%)		91.17(31/34)*	66.67(4/6)**	51.92(27/52)***	* *P*<0.05,** *P* = 0.154,*** *P* = 0.8

* drug-resistance group vs drug-sensitive group,

** drug-resistance group vs partially sensitive group,

*** partially sensitive group vs drug-sensitive group.

Patients were assigned to groups according to criteria from the NCCN guidelines 2012 (National Comprehensive Cancer Network). Based on these criteria, (1) *the chemotherapy-resistant group* included patients who had a clinical response to the initial TC chemotherapy, but experienced subsequent relapse, either in the late stage of chemotherapy or within 6 months after completion of chemotherapy; (2) *the partially chemotherapy-sensitive group* included patients who experienced ovarian cancer relapse within 6–12 months after completion of chemotherapy; and (3) *the chemotherapy-sensitive group* included patients who maintained a clinical response for ≥12 months. Factors diagnostic for ovarian cancer relapse included continuously increased CA125 levels, solid lesions identified by gynecological examination, tumors identified by imaging and signs of ascites. In accordance with the NCCN guidelines described above, ovarian cancer patients were assigned to either the chemotherapy-resistant group (n = 34), the partially chemotherapy-sensitive group (n = 6) and the chemotherapy-sensitive group (n = 52).

### Immunohistochemistry

Immunohistochemistry was used to analyze Lewis y antigen and CD44 expression levels. The mouse anti-Lewis y monoclonal antibody (clone A70-C/C8, 1∶160 dilution) and mouse anti-CD44 monoclonal antibody (clone F-4, 1∶200 dilution) were purchased from Abcam (Abcam Co, Cambridge, UK) and Santa Cruz Biotechnology, Inc. (Santa Cruz, CA, USA), respectively. The staining procedure was performed as described in the manuals for the SABC (Strept Avidin-Biotin Complex) and SP(streptavidin-perosidase) kits.

Tissue sections were considered positive if light brown granules were present in the cell membranes and cytoplasm. Low-power images of sections were scored based on the staining intensity, and no staining, light yellow staining, light brown and brown staining were scored as 0, 1, 2 and 3, respectively. Subsequently, a total of 5 high-power fields in series were selected from each slice for scoring of individual cells. The mean percentages of positively stained cells were then calculated for each field. Fields with less than 5% positive cells were scored as 0; 5% to 25% as 1; 26% to 50% as 2; 51% to 75% as 3; greater than 75% as 4. These two scores were then multiplied to yield the final score. A final score of 0 to 2 was considered (−); 3 to 4 was (+); 5 to 8 was (++) and 9 to 12 was (+++). Score≥3 tumors are considered positive for Lewis y antigen and CD44 in statistical analysis. Two independent observers scored each section to control for observer error.

### Multi-variable analysis

We next performed a binary logistic regression analysis (forward: conditional) considering age, surgical stage, grade, pathological subtype, metastasis of lymph nodes, residual tumor size and the expression of Lewis y antigen and CD44 served as co-variates and drug resistance or sensitivity as the dependent variable, to analyze factors associated with chemotherapeutic resistance in ovarian cancer patients.

### Comparison of survival rates

As of May 2012, which was 7 years after enrollment of the first patient and 3 years after enrollment of the last patient, the mean follow-up was 34.0 months. Over this time period, 26 of 34 patients in the chemotherapy-resistant group, 5 of 6 patients in the partially chemotherapy sensitive group and 13 of 52 patients in the chemotherapy-sensitive group died of ovarian cancer relapse or metastasis. To determine whether Lewis y antigen and CD44 expression were associated with ovarian carcinoma outcomes, we compared the survival rates of the three groups.

### Statistical Analysis

Positive ratio rates were evaluated using the *χ^2^* test, Correlation analyses were performed using the Spearman rank order correlation coefficient. Kaplan-Meier survival curves were plotted using cancer-related death as the endpoints and compared using a log rank test. Binary logistic regression was used for multivariate analysis. All statistical analyses were performed using SPSS V13.0 software (SPSS, Illinois, USA). A two-tailed *p* value test was used in all analyses; a *p*-values less than 0.05 were considered statistically significant.

## Results

### Lewis y antigen and CD44 expression levels in ovarian cancer tissues from patients with varying sensitivity to chemotherapy

**Figure 1:**
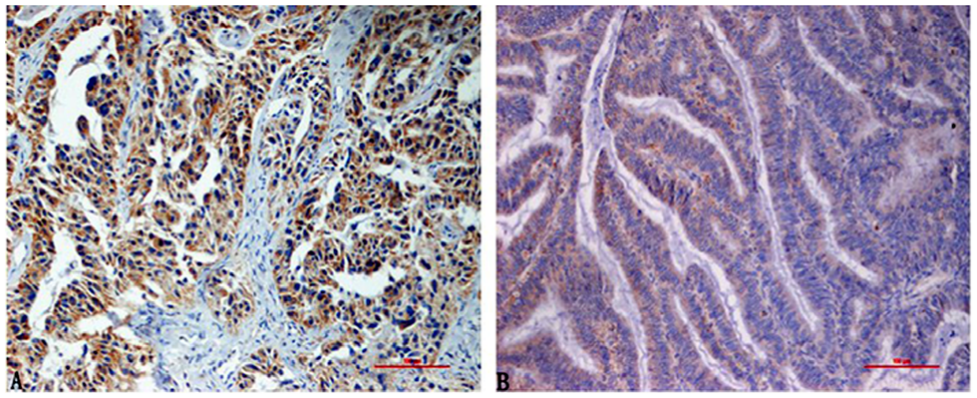
The expression of Lewis y in ovarian carcinoma tissues, the molecule was present primarily in the cell membrane, but also in the cytoplasm. (A chemotherapy-resistant group; B chemotherapy-sensitive group. ×200).

Staining for Lewis y antigen revealed that the molecule was present primarily in the cell membrane, but also in the cytoplasm (**Figure 1**). The rates of positive expression of Lewis y antigen in the drug-resistant, partially sensitive and sensitive groups were 91.17%, 55.00% and 61.54%, respectively (**Table 1**). The rate of positive expression of Lewis y antigen in the drug-resistant group was significantly higher than in the partially sensitive group and sensitive group (both *p*<0.05). No significant difference in expression of the Lewis y antigen was observed between the partially sensitive and sensitive groups (*p = 0.915*).

**Figure 2:**
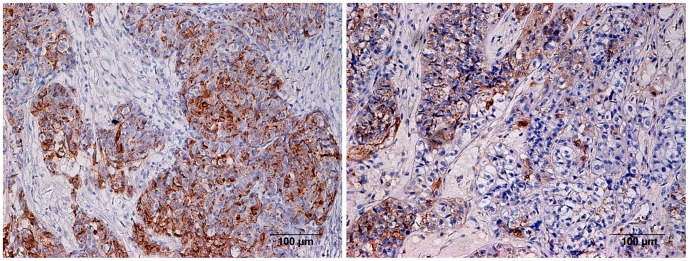
The expression of CD44 antigen in ovarian carcinoma tissues, CD44 primarily localized to the cell membrane, but was also observed in the cytoplasm and in the interstitial spaces outside of tumor cells (A chemotherapy-resistant group; B chemotherapy-sensitive group. ×200).

CD44 was also primarily localized to the cell membrane, but was also observed in the cytoplasm and in the interstitial spaces outside of tumor cells (**Figure 2**). The rates of positive expression of CD44 in the drug-resistant, partially sensitive and sensitive groups were 91.17%, 66.67% and 51.92%, respectively (**Table 1**). The rate of expression of CD44 in the drug-resistant group was significantly higher than that in the sensitive group (0.01<*p*<0.05), but no significant difference was observed between the drug-resistant group and the partially sensitive group (*p = 0.154*).

### Lewis y antigen and CD44 expression levels in different subtypes of ovarian carcinoma

Of the 92 cases analyzed, 58 were cases of serous carcinoma (34 sensitive, including 4 partially sensitive, 24 drug-resistant), 8 were cases of mucinous carcinoma (4 sensitive, 4 drug-resistant), 4 were cases of endomerioid carcinoma (3 sensitive), and 6 were cases of clear cell carcinoma (5 sensitive, including 1 partially sensitive). The rates of positive expression of Lewis y antigen and CD44 were 91.17% and 95.83%, respectively, in the serous cancer drug resistant group, which were higher than those of the sensitive group (56.67% and 53.33%, respectively). The rates of positive expression of Lewis y antigen and CD44 in cases of clear cell carcinoma and endometrioid carcinoma were similar to those in the serous group, although the number of cases in these groups were small. The drug resistant and sensitive groups each had 4 cases of mucinous carcinoma, and no significant difference was observed in the intra-group rates of Lewis y antigen and CD44 positivity (**Table 2**).

**Table 2:** Lewis y antigen and CD44 molecule expression levels in different histotype of ovarian carcinoma.

Group	Lewis y antigen			CD44 molecule		
	Drug-resistance	Sensitive	*P* value	Drug-resistance	Sensitive	*P* value
Serous carcinoma	22/24, (91.67)*	17/30,(56.67)	0.0043	23/24, (95.83)	16/30, (53.33)	0.0005
Mucinous carcinoma	3/4, (75.00)	3/4, (75.00)	>0.05	3/4, (75.00)	3/4, (75.00)	>0.05
Endometrioid adenocarcinoma	1/1, (100.00)	1/3, (33..33)	<0.05	1/1, (100.00)	2/3, (66.67)	<0.05
Clear cell carcinoma	1/1, (100.00)	3/5, (60.00)	<0.05	1/1, (100.00)	2/5, (40.00)	<0.05

* All cases listed in the form of n/N (%).

### Correlation between Lewis y antigen and CD44 expression levels in ovarian cancer tissues

Analysis of 92 ovarian cancer tissue samples revealed that the rate of positive expression of Lewis y antigen in the drug resistant group was higher than that of the sensitive group. Furthermore, the expression of CD44 also showed similar trends in the two groups. Results from a Spearman rank correlation test demonstrated that Lewis y antigen expression was significantly positively correlated with CD44 expression (Spearman coefficient *r_s_* = 0.3455, *p*<0.05).

### Multi-variable analysis of factors associated with chemotherapeutic resistance in ovarian cancer patients

Results from binary logistic regression analysis surgical stage, residual tumor size and expression of CD44 and Lewis y antigen in ovarian carcinoma tissues were independent risk factors for chemotherapeutic drug resistance (**Table 3**).

**Table 3:** Multi-variable analysis of factors associated with chemotherapeutic drug resistance.

Type	*P* value	Hazard Ratio (95% CI)
Stage	0.035	2.556 (1.067∼6.123)
Residual Tumor Size	0.004	1.721 (1.185∼2.498)
CD44	0.006	2.949 (1.355∼6.416)
Lewis y	0.016	2.154 (1.156∼4.014)

### Comparison of ovarian cancer patient survival

**Figure 3:**
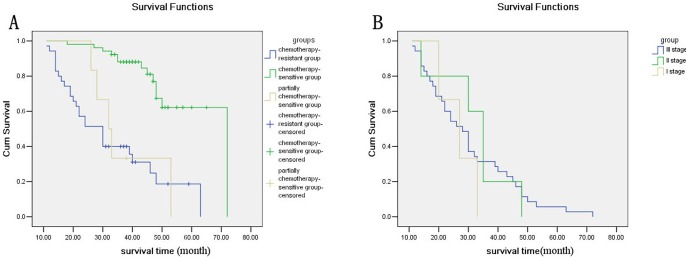
Kaplan-Meier curves of overall survival stratified by grouping, using cancer-related death as the endpoints (A curves of overall survival stratified by grouping, n = 92, p<0.001. B curves of deaths stratified by stage, n = 42).

Results from follow-up revealed that 26 patients (1 stage I, 2 stage II, 23 stage III; mean age, 56.07; median age, 56) in the chemotherapy-resistant group died of ovarian cancer relapse and metastasis; 5 patients (1 stage II, 4 stage III; mean age, 64.6; median age, 63) in the partially chemotherapy-sensitive group died, and 13 patients (2 stage I, 3 stage II, 8 stage III; mean age, 54.58; median age, 54) in the chemotherapy-sensitive group died. Overall survival was significantly shorter in the chemotherapy-resistant group (*p*<0.001, **Figure 3**) compared to the sensitive groups. However, as a greater percentage of deaths in the chemotherapy resistant group were from stage III cancers, we cannot conclusively state that glycosylated Lewis y antigen and CD44 are associated with survival outcomes.

## Discussion

Chemotherapy plays an important role in the management of epithelial ovarian cancer, but acquired resistance to chemotherapy strongly affects therapeutic efficacy and patient survival. CAM-DR is a complicated process involving interactions between adhesion molecules and their associated receptors. One such cell adhesion receptor is CD44, a widely-distributed transmembrane cell surface glycoprotein. CD44 has a complicated and variable structure due to alternative splicing at the transcriptional level and multiple post-translational modifications. CD44 is the major HA receptor, and interaction with HA is involved in tumor cell adhesion and migration [9]. Binding of CD44 to HA is regulated by multiple factors, of which glycosylation or glycosaminoglycan modification is the most significant. The molecular weight of unmodified CD44 is 37 kDa, but post-translationally modified CD44 ranges from 85 to 95 kDa [10]. Glycosylation is essential for the CD44-HA interaction, and different oligosaccharide modifications have different effects on CD44 function. Our preliminary studies have shown that di-fucosylated Lewis y antigen is a part of the composition of CD44 and increased levels of Lewis y antigen are associated with increased CD44-mediated ovarian cell adhesion (including adhesion to HA) and migration [6]. We also found that cell lines that highly express Lewis y antigen and CD44 exhibit increased resistance to chemotherapeutic drugs, such as carboplatin and paclitaxel [7], [8].

Based on our preliminary studies, we initiated the present study to investigate the correlation between expression of CD44 and its associated structural oligosaccharide Lewis y antigen and resistance to therapy and prognosis for ovarian cancer. Our results showed that the rate of CD44-positive expression in the chemotherapy-resistant group was significantly higher than that of the partially chemotherapy-sensitive group (91.17% vs. 66.67%; *p*<0.05) and that of the chemotherapy-sensitive group (51.92%; *p*<0.025). These data suggest that increased expression of CD44 is closely related to drug resistance of ovarian cancers, consistent with results from several previous studies [11], [12].

We next investigated the role of the Lewis y antigen structural motif in drug resistance of epithelial ovarian cancers. The rates of Lewis y antigen-positivity were 91.67%, 50.00%, and 61.54% in the chemotherapy-resistant, partially chemotherapy-sensitive, and chemotherapy-sensitive groups, respectively, with a significant difference between the former group and the latter two groups (0.005<*p*<0.01). Expression of Lewis y antigen and that of CD44 were positively correlated (*r_s_* = 0.3455, *p*<0.05). This observation is consistent with the hypothesis that fucosylation of CD44 is associated with drug resistance in ovarian cancers. Multi-factor logistic regression analysis further confirmed that expression of Lewis y antigen and CD44, surgical stage of ovarian cancer, and size of residual lesions were all independent factors associated with chemotherapeutic resistance. These data provide evidence supporting an important role of glycosylation in the process of development of drug resistance in tumor cells.

Studies have reported that glycosylation plays an important role in tumor cell drug resistance. For example, in oxaliplatin-resistant ovarian cancer cells (IGROV-1/OHP), N-glycosylation of the highly-expressed, ATP-binding transport proteins MRP1 and MRP4 is abnormally increased [13]. In oral squamous cell carcinoma cells (HSC-2) and intestinal adenocarcinoma cells (LS174T), inhibition of N-glycosylation using tunicamycin blocks the strong apoptotic activity of the prion protein PrPc [14]. Furthermore, the expression of α-1,6-fucosyltransferase has been reported to be increased significantly in mitoxantrone-resistant liver cancer cell strains (HLE-MIT) [15]. Together, these findings strongly implicate glycosylation plays an important role in drug resistance mechanisms across many types of cancer. Follow-up of 92 ovarian cancer patients in the present study revealed that the mortality rate of chemotherapy-resistant patients was significantly higher than that of chemotherapy-sensitive patients, However, as a greater percentage of deaths in the chemotherapy resistant group were from stage III cancers, we cannot conclusively state that glycosylated Lewis y antigen and CD44 are associated with survival outcomes.

In recent years, the mechanisms underlying the role of CD44-HA in drug resistance have been widely studied, but no uniform conclusion regarding the precise mechanisms has been reached. In vivo and in vitro studies conducted by Lakshman M et al [16], [17]. have shown that CD44 is involved in inhibition of etoposide-induced apoptosis in intestinal cancer cells. High expression of CD44 was associated with decreased expression of Caspase-3 and Caspase-9 and decreased phosphorylation of AKT, suggesting that CD44-mediated inhibition of apoptosis was regulated by mitochondrial signaling pathways. Results from a study by Bourgiugnon LY et al. [18] revealed that HA-bound CD44 activated RhoA and Rho kinase signaling, increased the serine/threonine phosphorylation of Grb2-associated binder-1 (Gab-1), activated PI3K, and promoted PI3K/Akt signaling, resulting in malignant progression of breast cancer. Steven J et al [19] found that the HA-CD44 interaction activated topoisomerase II (Topo-II) to decrease the cytotoxicity of etoposide in head and neck tumors. Bourguignon LY et al. [20] further showed that, in breast and ovarian cancer cell lines (MCF-7 and SKOV3), binding of CD44 to HA activated the Nanog-Stat3 signaling pathway to induce ankyrin binding to MDR1, forming a drug efflux channel that promoted the efflux of chemotherapeutic agents (doxorubicin and taxinol). The interaction between HA-CD44 and EGFR-ERK1/ERK2 was identified as a mechanism underlying failure of methotrexate plus cisplatin chemotherapy in head and neck squamous cell carcinoma (HNSCC) [21]. Marisa C [22] found that the invasive potential of Pgp-positive, drug-resistant melanocytoma cells was significantly increased over that of the parental cells. Importantly, CD44 colocalized with Pgp in the cytoplasm, where they interacted to activate the ERK1/2 and p38-MAPK signaling pathways, leading to an invasive and drug-resistant phenotype.

Our preliminary studies revealed that in transfected ovarian cancer cells highly expressing the Lewis y antigen (RMG-1-H cells), AKT phosphorylation levels were substantially increased in the absence of a change in total protein levels. Treatment with ZD1839 or Lewis y antibodies significantly decreased levels of phosphorylated AKT in transfected cells independently. Results from MTT assays showed that treatment with LY294002, a PI3K-specific inhibitor, inhibited the proliferation of transfected ovarian cancer cells highly expressing the Lewis y antigen, suggesting PI3K/AKT signaling is involved in Lewis y antigen-mediated proliferation of these cells [23]. Another study revealed that PI3K/AKT/mTOR signaling pathways serve as key regulators of platinum-based drug sensitivity of ovarian cancer cells [24]. The present study found that expression of CD44 and Lewis y antigen was increased in tissue sections from drug-resistant ovarian cancer patients. Furthermore, multi-variable analysis demonstrated that CD44 and Lewis y antigen expression were both independent risk factors for development of drug-resistant ovarian cancer. Together with results from our preliminary studies, these findings suggest that the Lewis y antigen, as an important component of the molecular structure of CD44, promotes proliferation and inhibits apoptosis of ovarian cancer cells, leading to drug resistance via activation of PI3K/AKT signaling pathways. Our preliminary studies also revealed that transfection with fucosyltransferase results in upregulated expression of Bcl-2, Topo-I, and Topo-II β in RMG-1-H cells, in addition to increased expression of the Lewis y antigen. Increased levels of the Lewis y antigen promoted the expression of Topo-I and Topo-II β through regulation of Bcl-2, resulting in enhanced DNA repair and inhibition of carboplatin-induced apoptosis. These findings indicate that the Lewis y antigen can also promote development of drug resistance in tumor cells through topoisomerase-dependent pathways [25]. Levels of the p38MAPK mRNA were also significantly increased by expression of the Lewis y antigen in transfected cells. In addition, levels of the p38MAPK and caspase-3 mRNAs were both increased in carboplatin-treated RMG-1-H cells, suggesting that Lewis y antigen-mediated apoptosis of ovarian cancer cells is associated with activation of the p38MAPK signaling pathway [26]. In conclusion, we propose that the Lewis y antigen, as a structural component of CD44, integrins α5β1 and αvβ3, as well as EGFR, play a role in various cell adhesion processes that mediate both cell adhesion and drug resistance [6], [27], [28], [29].

In the current study, we analyzed drug-resistant clinical ovarian carcinoma samples and found that that Lewis y antigen and CD44 were associated with ovarian carcinoma drug resistance. In light of our previous studies, we consider that the Lewis y antigen represents a key molecule involved in CAM-DR with potential as a novel anti-apoptotic target for the treatment of drug resistant ovarian carcinomas.
